# Ferroptosis surveillance: Insights from *in vivo* contexts

**DOI:** 10.70401/fos.2026.0021

**Published:** 2026-03-12

**Authors:** Alec J. Vaughan, Mario Palma, Jessalyn M. Ubellacker, Thales Papagiannakopoulos

**Affiliations:** 1 Department of Pathology, New York University Grossman School of Medicine, New York, NY 10016, USA.; 2 Vilcek Institute of Graduate Biomedical Sciences, New York University Grossman School of Medicine, New York, NY 10016, USA.; 3 Department of Molecular Metabolism, Harvard T.H. Chan School of Public Health, Boston, MA 02115, USA.; 4 Ludwig Center at Harvard, Harvard Medical School, Boston, MA 02115, USA.; 5 Laura and Isaac Perlmutter Cancer Center, New York University Langone Health, New York, NY 10016, USA.

**Keywords:** Ferroptosis, microenvironment, GPX4–FSP1 axis, *in vivo* modeling

## Abstract

Ferroptosis has emerged over the past decade as a compelling therapeutic avenue for cancer, prompting intense interest in strategies that selectively induce or inhibit this form of cell death. Although substantial progress has been made in identifying genes that regulate ferroptosis sensitivity and in developing small-molecule modulators, it remains unclear which molecular targets offer the greatest therapeutic potential in specific tissues and contexts. Here, we highlight fundamental differences between *in vitro* and *in vivo* ferroptosis modulation, with emphasis on the integration of different techniques, mouse models, and how the tumor microenvironment shapes two major ferroptosis surveillance pathways: glutathione peroxidase 4 and ferroptosis suppressor protein 1. We propose that integrating *in vivo* biological constraints and microenvironmental complexity is essential for the rational design and successful translation of ferroptosis-targeted therapies.

## 1. Introduction

The recognition of ferroptosis as a distinct modality of regulated cell death emerged from studies linking cysteine availability and glutathione peroxidase 4 (GPX4) activity to the control of lipid peroxidation^[1,2]^. At a molecular level, ferroptosis is governed by the interplay between lipid peroxidation-promoting pathways, often iron-catalyzed Fenton chemistry and enzymatic oxidation of polyunsaturated fatty acyl (PUFA)-containing phospholipids, and endogenous antioxidant defense mechanisms, mainly the Xc^−^-GSH-GPX4 and FSP1-CoQ10-NAD(P)H axes^[3–8]^. The imbalance of these ferroptosis surveillance systems leads to the accumulation of lipid peroxides within cellular membranes, ultimately compromising plasma membrane integrity and resulting in ferroptotic cell death^[3–8]^.

Early indications that various tissues like the brain and kidney are highly reliant on mechanisms to repair oxidative lipid damage emerged from seminal work from the Conrad group using conditional *Gpx4* knockout mice^[19]^. Further mechanistic insights into ferroptosis execution, regulation, and therapeutic targetability have emerged from *in vitro* and some *in vivo* studies, including cellular and biochemical models^[10–29]^. Recent advances in small-molecule inducers and inhibitors targeting these pathways have significantly advanced our understanding of ferroptosis, highlighting its potential as a strategy for inducing cell death in cancer^[30,31]^.

Ferroptosis is recognized as a form of metabolic cell death^[32,33]^. Several decades of cancer metabolism research have revealed that metabolic rewiring is a necessary step in tumorgenesis, but targeting these pathways has proven difficult due to tumor metabolic plasticity *in* vivo^[34,35]^. Since sensitivity to ferroptosis relies on several metabolic inputs, we propose that understanding how different *in vivo* metabolic environments in various cancer types impact ferroptosis will be crucial to the development and application of ferroptosis induction as a therapeutic approach.

## 2. *In Vitro* Versus *in Vivo* Ferroptosis Surveillance Systems

Ferroptosis surveillance has largely been defined through *in vitro* studies in which key protective pathways, most notably GPX4, system Xc^−^, and FSP1, are perturbed using CRISPR/Cas9 screens or pharmacologic inhibition^[6,7]^. In these settings, loss of surveillance is inferred when lipid peroxidation–driven cell death can be prevented by lipophilic antioxidants (such as α-tocopherol), iron chelators (Deferoxamine, Deferiprone), or synthetic radical-trapping antioxidants like liproxstatin-1 (LIP-1) or ferrostatin-1^[5,6]^. These rescue criteria have become foundational to the field, but most of our mechanistic understanding of ferroptosis surveillance derives from cells grown under standard *in vitro* conditions, in the absence of *in vivo* physiological conditions.

There is growing interest in targeting ferroptosis for cancer therapy, which has expanded efforts to define how ferroptosis surveillance operates *in vivo.* Emerging work shows that surveillance mechanisms behave differently across tissues and microenvironments, often diverging sharply from *in vitro* expectations. System Xc^−^ dependency, for example, varies substantially between tumor types and even within the same cancer across anatomic contexts. In some models, inhibition of system Xc^−^ robustly sensitizes tumors to ferroptosis, whereas in others it has little effect, reflecting microenvironmental constraints on cystine availability and glutathione metabolism^[21,36–40]^. GPX4 dependency is similarly context-dependent. Studies comparing blood and lymphatic environments demonstrate that melanoma cells rely on GPX4 in the oxidative bloodstream but become unexpectedly GPX4-independent in the lymphatic niche^[41]^, where FSP1-dependency can dominate. *Ex vivo* analyses reinforce this point: melanoma cells freshly isolated from lymph nodes show greater resistance to GPX4 inhibitors (RSL3, ML-210) or system Xc^−^ blockade (Erastin-2) than matched cells from subcutaneous primary tumors^[41]^.

Recently, several small-molecule inhibitors targeting FSP1 have been developed^[17,24,42,43]^. However, as with FSP1 genetic loss, FSP1 inhibitors alone are often insufficient to induce ferroptotic cell death *in vitro,* requiring co-administration with either genetic or pharmacologic GPX4 or Xc^−^ inhibition for efficacy^[30]^. Notably, two recent studies from our groups revealed that FSP1 is required for *in vivo* tumor growth and metastasis^[44 45]^.

The Papagiannakopoulos lab demonstrated that genetic loss-of-function of *Gpx4* and *Fsp1* in Kras-driven genetically engineered mouse model (GEMM) of lung adenocarcinoma (LUAD) led to dramatic suppression of lung tumor growth^[44]^. Consistent with previous studies, *Fsp1* knockout in LUAD or pancreatic ductal adenocarcinoma (PDAC) cells did not affect viability *in vitro,* and the induction of ferroptosis was only observed with *Gpx4* inhibition^[44]^. In both GEMM and orthotopic transplantation experiments, *Fsp1* knockout tumors displayed increased lipid peroxidation and decreased tumor growth, which was rescued by ferroptosis inhibition via LIP-1 treatment, dietary vitamin E, and genetic deletion of Acsl4. These studies suggest that *Fsp1* loss alone induces ferroptosis *in vivo.* Importantly, pharmacological inhibition of FSP1 (icFSP1) *in vivo* induced ferroptosis and significantly suppressed tumor growth^[44]^.

In a complementary study, the Uberllacker lab demonstrated that melanoma colonization of lymph nodes (LNs) induces a shift in the GCLC-GSH-GPX4 and FSP1-CoQ10-NAD(P) axes, resulting in reduced dependency on GPX4 and increased reliance on FSP1 in LN metastatic cells^[45]^. Consistent with the findings from the previous studies, *in vitro* co-targeting of FSP1 alongside other small molecule inducers, such as BSO to inhibit GCLC, significantly impaired the viability of LN metastatic lines, both under normoxic conditions (21% O_2_, standard tissue culture) and hypoxia (1% O_2_, recapitulating the LN microenvironment)^[45]^.

However, in the *in vivo* context, both genetic ablation and pharmacological inhibition of FSP1 alone (via viFSP1 and FSEN1) markedly suppressed melanoma growth within LNs but failed to affect subcutaneous (s.c.) tumor growth, emphasizing a context-dependent regulation of ferroptosis sensitivity determined by the tumor microenvironment^[45]^. Furthermore, studies using LN metastatic melanoma lines with either wild-type or *Fsp1* KO confirmed that FSP1 is specifically required for LN metastasis, while dispensable for hematogenous metastasis^[45]^. Conversely, GPX4 is essential for melanoma survival in the bloodstream but not within LNs^[41]^, whereas FSP1 is required in LNs but not during hematogenous spread^[45]^. Together, these findings reveal a finely tuned, microenvironment-specific dependency on distinct ferroptosis protective pathways *in vivo*.

Collectively, this work emphasizes the current understanding of the *in vivo* relevance, temporal and spatial regulation, and therapeutic potential of FSP1, positioning FSP1 as a key ferroptosis vulnerability. The observed differences in FSP1 dependence *in vivo* versus *in vitro* suggest the importance of incorporating and considering *in vivo* ferroptosis features into *in vitro* systems, as outlined in the following sections.

## 3. Modeling Ferroptosis Surveillance *in Vitro*

As discussed above, ferroptosis is regulated differently *in vitro* and *in vivo,* creating substantial challenges for both mechanistic analysis and translational application. When possible, mirroring key elements of *in vivo* ferroptosis surveillance within *in vitro* systems will likely enhance the *in vivo* translatability of ferroptosis-targeted strategies.

A major limitation in the *in vivo* setting is the lack of tools that directly report ferroptotic cell death or monitor GPX4 and FSP1 dynamics. Commonly used assays, such as 4-HNE staining, TUNEL, or bulk lipid-peroxidation measurements, are indirect and cannot definitively attribute cell death to ferroptosis. More specific methods, including oxylipidomics or newly developed ferroptosis sensitive reporters, are needed to accurately assess ferroptosis *in vivo.* Furthermore, genetically encoded sensors capable of tracking GPX4 or FSP1 activity, localization, or stability would greatly advance the mechanistic resolution of these pathways in physiological contexts. Until such tools become available, validation using ferroptosis-selective inhibitors (for example, liproxstatin-1 or vitaminE) remains essential.

Therefore, *in vitro* assays remain indispensable for progressing ferroptosis research. However, it is critical to recognize that conventional two-dimensional cultures maintained in standard culture conditions lack the architectural, metabolic, and microenvironmental complexity that shapes ferroptosis susceptibility *in vivo.* Thus, where possible, incorporating advanced *in vitro* systems, including three-dimensional spheroids, organoids, engineered extracellular matrices, tailored culture media (such as TIFM, HPLM, or other next-generation formulations), and co-culture models, will allow for recapitulation of spatial and microenvironmental cues that can influence GPX4- and FSP1-dependent protection.

Nutrient levels in standard culture media differ markedly from those in tumor microenvironments^[46]^. For instance, DMEM and RPMI contain ~5-fold higher cystine than tumor interstitial fluid^[46]^, potentially increasing GPX4 dependence. Selenium concentrations also vary between serum batches and media^[47]^, which may influence GPX4 expression and ferroptosis sensitivity. Likewise, lipid supply is largely dictated by fetal bovine serum, which poorly reflects *in vivo* lipid composition and is highly batch-variable. Additionally, some 3D culture systems (organoids) require antioxidant supplementation, which can further influence ferroptosis sensitivity^[48]^. Thus, careful selection and reporting of culture media are essential in the field, as ferroptosis phenotypes observed *in vitro* may reflect nutrient-replete, non-physiological conditions that mask or distort vulnerabilities present *in vivo.*

Additionally, incorporating genetic models and using orthotopic or immunocompetent systems is strongly recommended, as these approaches increase the physiological relevance of ferroptosis studies by capturing variable factors that remain difficult to control *in vitro.* Integrating these complementary approaches, with clear awareness of their respective strengths and limitations, will improve the translational value of ferroptosis research and strengthen both *in vitro* and *in vivo* experimental strategies (Table 1).

## 4. Tumor Microenvironment and Ferroptosis Modulation

The TME is a dynamic and metabolically diverse niche that profoundly influences cancer progression and therapy response^[50]^. Consequently, accurate *in vitro* modeling of ferroptosis requires explicit incorporation of key TME features, which are essential for interpreting ferroptotic responses within physiological contexts. In the following sections, we examine how variations in lipids, oxygen levels, amino acids, and the immune cells shape the regulation and therapeutic vulnerability of ferroptosis *in vivo.* We also highlight why these parameters must be integrated into the ferroptosis field, just as they are already fundamental considerations in other fields such as cancer metabolism.

### 4.1 Lipids

The mechanism of ferroptosis induction, oxidation of PUFAs in cellular membranes, is tightly linked to the availability of PUFAs in cellular membranes. This is best shown through CRISPR genetic screens, where ACSL4 loss, leading to decreased long chain PUFA species in PL membranes, has consistently been shown to lead to greater resistance to ferroptosis induction *in vitro* and *in* vivo^[20,51–55]^. However, when available ferroptosis CRISPR screens were analyzed, ACSL4 was shown to only lead to resistance in screens where GPX4 was inhibited and not when cysteine was depleted or uptake was blocked via erastin family members^[53]^. As mentioned above, we found that *Acsl4* KO rescued the growth of *Fsp1* KO LUAD tumors, suggesting that in the context of the lung, Acsl4 is necessary for the induction of ferroptosis^[44]^.

Later, fundamental work by several groups identified the action of ACSL3 in mediating resistance to ferroptosis via incorporation of MUFA species that limit the availability of ferroptosis substrate PUFA species^[41,54]^. Importantly, analysis of lipidomics of serum and tissue culture media revealed that lipid quantities in media were frequently an order of magnitude lower than in serum. Indeed, when media was supplemented with physiological levels of oleic acid, this protected cells *in vitro* from ferroptosis and was dependent on ACSL3^[41]^. *In vivo,* pretreatment with oleic acid increased the ability of cells to metastasize through blood, and loss of ACSL3 decreased metastasis through the lymph^[41]^. Together, these data indicate that the availability of MUFA lipid species in the tumor environment provides a context where the availability of specific lipids protects tumor cells from ferroptosis and thus is an important consideration for ferroptosis-targeting *in vivo.*

As highlighted above the availability of pro-ferroptotic lipid species can significantly impact the induction of ferroptosis both *in vitro* and *in vivo.* This was recently highlighted in an elegant study, which used an inducible system to deplete GPX4 from cells grown in 2D, 3D spheroids, and as subcutaneous tumors *in* vivo^[55]^. Surprisingly, GPX4 loss after tumor initiation had no effect on the growth of the tumors, 3D spheroids were largely resistant to ferroptosis, while 2D cultures were sensitive. These differences were not due to increased expression of anti-ferroptotic genes, but to stark lipidomic differences when comparing cells grown in the various conditions. They found that they could resensitize 3D cultures to GPX4 inhibition by supplementing exogeneous PUFA species. These data illustrate that *in vitro* ferroptosis phenotypes may not always be replicated *in vivo* due to lipidomic differences between model systems. These data also suggest that 3D cultures may more accurately recapitulate some of the lipidomic environment found *in vivo,* although the tumors were grown as subcutaneous xenografts, outside of the respective TME from which they were derived.

Several groups have also recently reported that intermediates in cholesterol biosynthesis, including squalene and 7-DHC, can directly suppress ferroptosis^[18,27,28]^. Loss of DHCR7 in pre-clinical models of Burkitt’s lymphoma led to an increase in 7-DHC levels, tumor growth, and a decrease in overall mouse survival^[27]^. Other groups have recently found that PDAC tumors with elevated levels of cholesterol biosynthesis have increased DCHR7 expression and 7-DHC levels that correlate with increased lung metastatic colonization^[56]^. Further, DHCR7 mutations reported in Burkitt’s lymphomas are associated with increased 7-DHC levels, suggesting potential relevance of 7-DHC-mediated ferroptosis protection in human disease^[57]^. Taken together, these data indicate that tumors can rely on elevated levels of cholesterol biosynthesis intermediates to inhibit ferroptosis, which can increase tumor growth or organ-specific metastasis.

### 4.2 Oxygen levels

Oxygen levels in healthy mammalian tissues typically range from 0% to 19%^[58]^, compared to the standard cell culture conditions (21% O_2_). Low oxygen levels in tissues, a condition known as hypoxia, can impact numerous cellular functions across diverse cell types^[59]^. Hypoxia is associated with both physiological as well as pathological conditions, including solid tumors and ischemia^[60]^.

Recent studies show that differences in oxygen levels play a pivotal role in the regulation of ferroptosis. In melanoma, low oxygen levels induce the ubiquitination and proteasomal degradation of GPX4, as well as a reduction in GSH levels^[45]^. These changes ultimately impact the efficacy and targetability of the GSH-GPX4 axis, particularly in LNs^[45]^, where oxygen concentrations range between 1–3%^[61]^. Similar findings have been observed in other cell lines^[62]^.

In addition, HIFs dependent and independent mechanisms have been associated with modulating ferroptosis. Recently, a HIF-independent mechanism showed that hypoxia suppresses ferroptosis by inhibiting the oxygen-dependent histone demethylase KDM6A, reducing expression of lipid metabolic enzymes such as ACSL4 and ETNK1, and thereby rewiring the cellular phospholipid profile to promote ferroptosis resistance^[63]^. Similarly, the activity of SCD1, and thus the levels of MUFA lipid species like oleic acid, are oxygen dependent^[64]^. In this manner, oxygen levels regulate the availability of anti-ferroptotic lipids, impacting ferroptosis sensitivity^[67,68]^.

Therefore, oxygen has emerged as another critical modulator of ferroptosis, especially in *in vivo* contexts where its levels are modulated by various factors and differ among tissues including solid tumors, contrasting with the more uniform conditions of *in vitro* systems.

### 4.3 Amino acids

Amino acids (AAs), which are the basic units of proteins and essential biomolecules, are critical for the maintenance of homeostasis^[67]^ and also play a role in regulating ferroptosis^[68,69]^. They contribute to maintaining iron, lipid, and redox balance^[68,69]^. However, the effects of amino acids on ferroptosis are complex and can vary depending on the context. Gln/Glu, Arg, and Lys are linked to promoting ferroptosis, while Cys, Met, Gly, Ser, Trp, Thr, and BCAAs are considered potential inhibitors of ferroptosis^[68,69]^.

Cysteine is a central metabolic determinant of ferroptosis, serving as the key precursor for glutathione (GSH) synthesis and antioxidant defense^[70]^. However, most mechanistic insights into cysteine-dependent ferroptosis have arisen from *in vitro* systems, where cystine availability is artificially defined and lacks the complexity of the tumor microenvironment where it is biologically relevant^[71]^. Previous studies show that PDAC relies on cystine uptake via system Xc^−^ to suppress ferroptosis through glutathione and coenzyme A synthesis^[38]^. Deleting the system xc− subunit *Slc7a11* or treating with cyst(e)inase induced lipid oxidation and inhibited PDAC growth *in* vivo^[38]^, revealing a promising therapeutic strategy.

Beyond sulfur-containing amino acids, the selenium–selenocysteine axis adds an additional regulatory layer to ferroptosis sensitivity *in vivo.* Selenocysteine is required for the maturation and catalytic activity of GPX4^[72]^. Thus, selenium availability, its metabolic conversion to selenide, and selenocysteine biosynthesis directly influence GPX4 function and shape tissue-specific susceptibility to ferroptosis^[73–75]^. Variations in dietary selenium, tissue uptake mechanisms, or microenvironmental selenium pools can therefore modulate ferroptotic responses in ways that may not be recapitulated in standard culture media.

AAs concentrations often differ significantly between standard *in vitro* culture conditions and those found *in vivo* within various tissues or cancers. These differences can impact the regulation of ferroptosis, potentially leading to discrepancies between *in vitro* and *in vivo* experiments. Therefore, more in-depth studies are needed to understand how *in vivo* amino acid environments influence ferroptosis compared to traditional cell culture models.

### 4.4 Immune cells

The regulation, execution, and targetability of ferroptosis have been primarily investigated in a cell-autonomous manner, whereas non-cell autonomous mechanisms of ferroptosis regulation have largely not been elucidated. Different groups disagree on the effect of ferroptotic death on immune cell activation^[76–78]^. For example, recent work showed that in a mouse model of lung cancer, loss of Gpx4 after tumor initiation paradoxically increased the tumor growth^[79]^. Mechanistically, this was due to an increase in secreted oxidized lipid species, which increased T cell exhaustion and impaired CD8^+^ cytokine production. However, when cell lines derived from primary tumors were grown subcutaneously, Gpx4 KO led to decreased tumor growth, which could be rescued by LIP-1. Further work using genetic models of ferroptosis induction and ferroptosis inhibitors in tumors is needed to clearly identify the impact of ferroptosis on the immune response.

As another example, T cell specific Gpx4 KO had no effect on thymic development, but prevented antigen-specific clonal expansion in response to viral infection due to an induction of ferroptosis^[80,81]^. Thus, potential GPX4 targeting therapies may limit the anti-tumor immune T cell response. However, this sensitivity does not extend to CD4^+^ Tregs, in which Treg specific Gpx4 loss increased the anti-tumor immune response and decreased tumor growth^[82]^. There is evidence that pathologically activated neutrophils (PMN) cells undergo ferroptosis in the tumor environment, and that this contributes to immune suppression and tumor growth^[83]^. However, other data suggests that in tumor infiltrating neutrophils, high levels of itaconate produced by Acodl inhibit KEAPl-mediated degradation of NRF2 and thus protects these cells from ferroptosis, increasing breast cancer metastasis^[84,85]^.

Tumor associated macrophages have well described roles in regulating local tissue metabolism and support the growth of tumor cells by secreting various metabolites including iron and selenium^[86]^. Interestingly, pro-inflammatory (Ml-like) macrophages have been shown to be more resistant to ferroptosis than immunosuppressive (M2-like) populations^[87]^. These immune cell subsets, possessing differential sensitivity to ferroptosis, could contribute to anti-tumor immune responses with systemic administration of ferroptosis inducers. Indeed, whether FSP1 or other ferroptosis pathways have roles in immune cells remains an area of active investigation and is essential to determining whether inducing tumor cell ferroptosis can be a viable therapeutic strategy. *Ex vivo* culture of primary immune cells within these contexts offers a powerful intermediate system that preserves key features of the *in vivo* microenvironment while allowing controlled perturbations, helping to bridge mechanistic insights from *in vitro* models with physiologic immune behavior.

## 5. Considering Tissue-Specific Contexts for Modeling and Targeting Ferroptosis *in Vitro*

**Figure 1.**
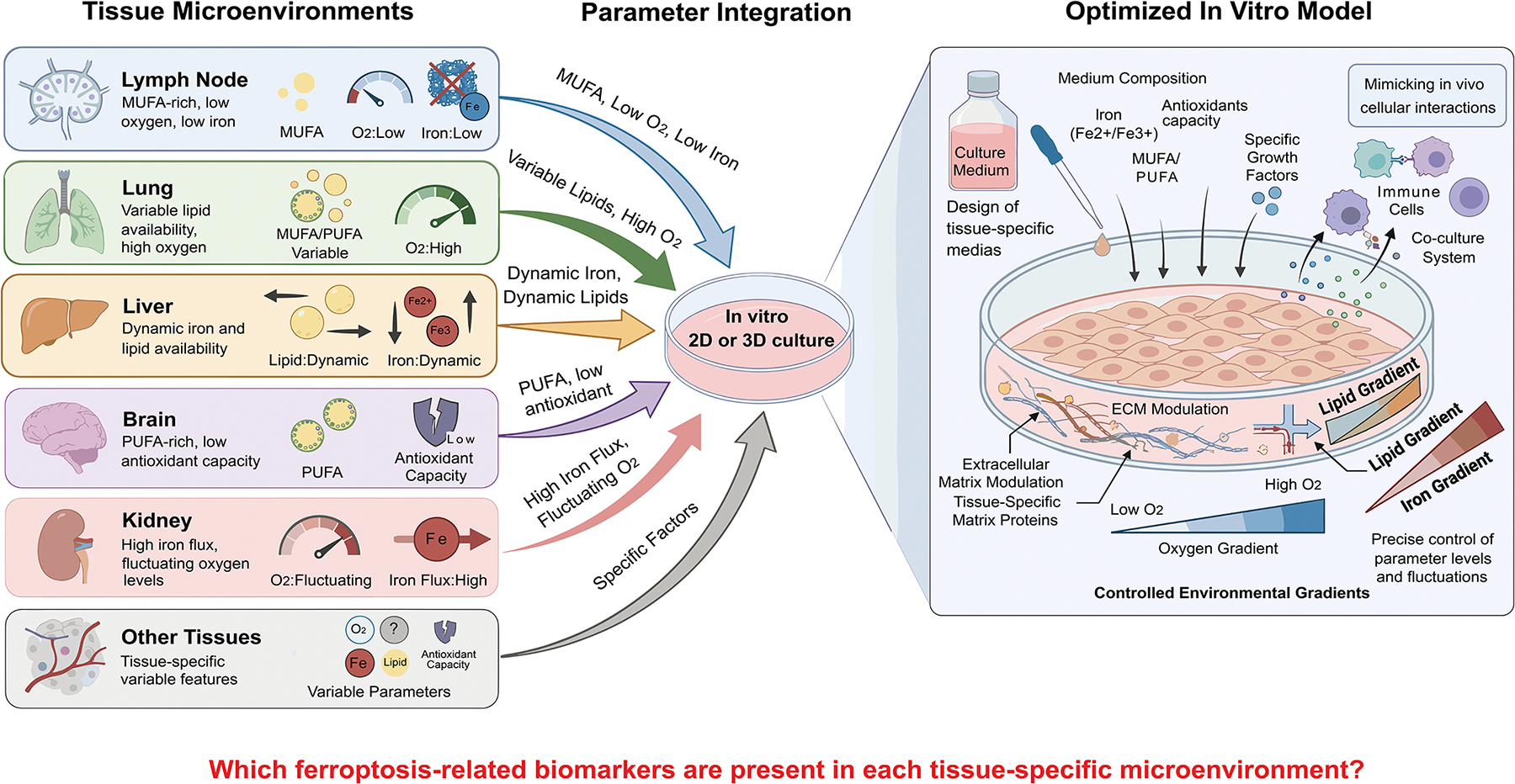
Schematic of organ-specific features relevant to ferroptosis that should be incorporated into in vitro models. Each tissue exhibits a distinct combination of lipid availability, redox buffering capacity, iron handling, oxygen tension, and immune activity. Accounting for these parameters, through the design of tissue-specific media, modulation of extracellular matrix composition, and control of gradients or levels of oxygen, lipids, iron, antioxidants, and immune interactions, can improve the translation of ferroptosis from physiological settings to controlled experimental systems. At present, many ferroptosis-associated features within the tumor microenvironment remain context dependent and lack sufficient specificity or validation to function as universal biomarkers *in vivo*; this challenge remains an important area of investigation in the field. Created with FigureLabs.

Given the emerging understanding that ferroptosis regulation *in vivo* is tightly shaped by microenvironmental conditions, it is expected that ferroptosis sensitivity varies markedly across organs. Each tissue presents a distinct combination of lipid availability, redox-buffering capacity, iron handling, oxygen tension, and immune activity. Consequently, accounting for these tissue-specific microenvironmental factors is essential when modeling ferroptosis *in vitro,* as they fundamentally influence ferroptotic responses and determine the translational relevance of experimental findings. Incorporating organ-specific parameters into *in vitro* systems enables more accurate extrapolation of ferroptosis biology from physiological settings to controlled experimental models (Figure 1).

Below, we highlight key determinants that influence ferroptosis across different organs:

### Lymph nodes.

Lymph nodes form a metabolically protective niche enriched in oleic acid^[41]^, low oxygen tension^[61]^, specialized lipid trafficking^[41]^, and elevated GSH^[41]^, all of which modulate ferroptosis. Our work shows that tumor cells in this environment shift from GPX4 toward FSP1 dependence^[45]^, reflecting how lymphatic conditions reshape ferroptosis surveillance. *In vitro* systems intended to approximate lymphatic ferroptosis sensitivity could therefore consider low-oxygen conditions, oleic-acid–rich lipid availability, and relevant immune co-culture components, among other factors.

### Lungs.

The lung is an oxygen-rich, lipid-dense, immune-dynamic organ where oxidative pressure is high^[88–90]^. Our findings that both *Gpx4* and *Fsp1* are required for LUAD growth indicate that lung tumors must activate strong ferroptosis surveillance to survive^[44]^. *Lung-mimicking in vitro* systems could therefore incorporate high oxygen tension, abundant lipid substrates, and air–liquid interface or alveolar-like models to capture the oxidative and immunologic demands of lung tissue.

### Liver.

As the central hub of iron^[91]^ and lipid metabolism^[92]^, the liver is particularly susceptible to ferroptosis under conditions of steatosis, inflammation, or iron overload, which can weaken immune surveillance defenses^[93]^. *In vitro* liver models should incorporate variable iron exposure, altered lipid states, or steatosis-like conditions to reflect physiologic or pathologic liver environments when probing GPX4 and FSP1 dependencies.

### Brain.

The brain’s high PUFA content^[94,95]^, abundant iron^[96]^, and comparatively limited antioxidant capacity^[97]^ make it intrinsically vulnerable to ferroptosis. Oxygen gradients, neuronal lipid composition, and the blood–brain barrier strongly shape ferroptosis surveillance, with GPX4 playing a particularly critical role^[98]^. Brain-relevant *in vitro* models should consider PUFA-rich lipid environments, controlled oxygen gradients, and, when possible, organotypic or barrier-mimicking structures.

### Kidney.

Renal tubular cells experience fluctuating oxygenation^[99]^ and intense redox stress^[100]^, making them highly sensitive to ferroptosis during acute kidney injury^[101,102]^. Kidney-mimicking *in vitro* systems should incorporate dynamic oxygen changes, iron flux, and high mitochondrial activity to reflect these pressures.

### Other tissues.

Organs such as heart, spleen, and skin each maintain unique ferroptosis surveillance landscapes shaped by their oxygenation profiles, immune composition, nutrient availability, and lipid architecture.

These differences highlight that studying ferroptosis *in vitro* should begin with an explicit question: which microenvironment is being modeled? Tailoring oxygen levels, lipid substrates, iron availability, and cell-cell interactions to the tissue of interest will improve the accuracy and translational relevance of ferroptosis studies (Figure 1).

## 6. Conclusions and Future Directions

Taken together, emerging evidence demonstrates that ferroptosis is regulated, and can be therapeutically targeted in distinct ways *in vitro* versus *in vivo.* Here, we have summarized the evidence supporting these differences, highlighted the value of integrating complementary *in vitro* and *in vivo* approaches, and emphasized the importance of accounting for microenvironmental factors that influence ferroptotic responses. We further emphasize how tissue-specific microenvironments may profoundly remodel ferroptosis-surveillance mechanisms and, in turn, shape ferroptosis vulnerabilities across physiological and pathological settings through differences in lipid availability, redox-buffering capacity, iron handling, oxygen tension, and immune activity. These insights make clear that tissue context must be considered to fully understand how ferroptosis is modulated *in vivo* and to accurately recapitulate these conditions *in vitro.*

Despite substantial progress, establishing a reliable foundation for ferroptosis-based therapeutic strategies will require addressing several outstanding questions:

- To what extent do *in vitro* and specific *in vivo* microenvironment conditions alter the dependency of specific ferroptosis surveillance systems? How do they influence FSP1- and GPX4-dependent protection?
- How do ferroptosis surveillance mechanisms operate across different tissues? Across primary or metastatic tumor microenvironments?
- Which ferroptosis-related biomarkers are present in each tissue-specific microenvironment?
- How should the field reconcile findings pertaining to ferroptosis-targeting from *in vitro* versus *in vivo* contexts?
- What criteria should define bona fide ferroptosis in a living organism?
- How do metabolic, lipidomic, and immunologic features of the microenvironment cooperate to suppress or permit ferroptotic death *in vivo*?

Progress in the field will require the development of robust *in vitro* models of ferroptosis that incorporate key microenvironmental factors, as well as systematic evaluation of diverse *in vivo* systems that integrate genetic, pharmacologic, and microenvironmental perturbations. In particular, combining genetic manipulation with well-defined chemical agents tested in parallel *in vitro* and *in vivo* will be critical for distinguishing context-dependent regulatory mechanisms. Additionally, dietary and even genetic factors have recently been shown to influence ferroptosis susceptibility^[98,103–105]^. Therefore, consideration of how such inter-individual biological differences shape the tumor microenvironment and subsequent ferroptosis susceptibility will enable a more integrated understanding of the role of ferroptosis in cancer. Future efforts should be accompanied by a rigorous assessment of how ferroptosis is defined and how it is regulated at the organelle and molecular levels across different tissues and microenvironmental contexts, analogous to the frameworks that have guided progress in the cancer metabolism field. Such an integrated approach will be essential for fully elucidating the biological relevance of ferroptosis and determining its real therapeutic targetability in cancer.

## Figures and Tables

**Table 1.** Overview of *in vivo* and *in vitro* models to study ferroptosis, highlighting key features, limitations, advantages and disadvantages.

Model System	What It Captures Well (Strengths)	What It Fails to Capture (Limitations)	Advantages	Disadvantages

**2D monolayer culture**	• Genetic dependencies • Rapid, high-throughput perturbations • Clean biochemical readouts	• No oxygen, lipid, nutrient, or iron gradients • No immune or stromal interactions • Overestimates GPX4 dependency • Misses FSP1-dependecy in some contexts	• Easy, inexpensive • Highly reproducible • Ideal for mechanistic screens	• Least physiologic • Artifacts from high oxygen and lipid composition
**3D spheroids**	• Hypoxia and nutrient gradients • Limited diffusion of lipid/iron • Partial recapitulation of tumor architecture	• Still lacks stromal/immune components • Limited extracellular matrix complexity • Cannot capture organ-specific lipid environments	• Better approximation of microenvironmental pressures • Maintains gradients relevant for ferroptosis	• Variable size and reproducibility • Some organoid culture medias contain antioxidants that may influence ferroptosis susceptibility
**Co-culture systems (tumor + stromal/immune cells)**	• Immune-driven modulation of ferroptosis surveillance • Cytokine and ROS cross-talk	• Still lacks full tissue architecture • Oxygen and lipid environments simplified	• Useful for evaluating the immune role in ferroptosis regulation	• Complex interpretation • Requires optimization for each co-culture
**Organoids**	• Tissue-specific architecture • Endogenous lipid composition • More realistic GPX4/FSP1 hierarchy • Better recapitulation of redox environments	• Lacks circulation, immune context • Limited iron flux modeling • Microenvironment still simplified	• Captures organ-level ferroptosis surveillance • Flexible for drug testing	• Expensive, technically demanding • Limited scalability
**Mouse orthotopic tumor models**	• Native microenvironment (oxygenation, lipids, iron) • Tissue-specific pressures on GPX4/FSP1 • Appropriate nutrient and metabolic gradients	• Hard to interpret cell-intrinsic vs microenvironmental effects • Readouts often indirect	• High physiological accuracy • Captures tissue-specific ferroptosis surveillance directly	• Expensive, time-consuming • *In vivo* analysis of ferroptosis requires specialized tools
**Immunocompetent mouse models**	• Complete immune-tumor interaction • Ability to study immune-regulated ferroptosis surveillance • Accurate lipid, iron, and stromal environments	• Human cancer lines not compatible • Immune variation adds complexity • Harder to isolate tumor-intrinsic effects	• Most physiologic ferroptosis surveillance • Ideal for therapy testing (e.g., GPX4/FSP1 inhibitors)	• Difficult to dissect mechanisms cleanly • High variability • Regulatory/ethical considerations

## Data Availability

Not applicable.
